# Primary diffuse large B-cell lymphoma of adrenal gland: A case report

**DOI:** 10.1097/MD.0000000000038298

**Published:** 2024-06-14

**Authors:** Yong Ou, Kai Wang, Ting-Yu Jia, Zhi-Gang Chen, Hua-Kang Wang, Ming-Xian Chen, Hua Yang, Zhi Liao, Hou-Chuan Chen, Zhong Zheng

**Affiliations:** a Department of Urology, Xichang People’s Hospital, Xichang, Sichuan, P.R. China.

**Keywords:** adrenal glands, diffuse large B-cell, lymphoma

## Abstract

**Introduction:**

Most adrenal tumors are benign and primary adrenal malignancies are relatively rare. Primary adrenal lymphoma (PAL) is a very rare and highly aggressive malignant tumor with unknown etiology, atypical clinical symptoms, nonspecific imaging manifestations, difficult disease diagnosis and poor prognosis.

**Case report:**

This case report details a 42-year-old woman who was admitted to the hospital with a 1-year-old bilateral adrenal mass and 1-month-old left upper abdominal pain. Enhanced CT of the abdomen showed a right adrenal nodule and a large occupying lesion in the left adrenal region, with a high probability of pheochromocytoma. Intraoperatively, a huge tumor measuring about 12*12*10 cm was found in the left adrenal region, infiltrating the left kidney, spleen and pancreatic tail. Postoperative pathology: lymphocytes were found in the renal capsule and subcapsule, lymphocytes were found in the pancreas; lymphocytes were found in the spleen. Consider a tumor of the lymphohematopoietic system, possibly lymphoma.

**Conclusion:**

This case demonstrates that primary adrenal diffuse large B-cell lymphoma (PADLBCL) is highly aggressive, has a poor prognosis, is prone to recurrence, has poor therapeutic outcomes, and is difficult to diagnose. Clinicians should consider the possibility of PADLBCL when encountering huge adrenal-occupying lesions and consider chemotherapy before surgery. Reducing the tumor size before surgery is a more favorable therapeutic approach, thus prolonging the patient life and improving the quality of survival.

## 1. Introduction

Lymphoma is a malignant tumor originating from lymph nodes or lymphatic tissues. The first symptom is painless enlargement of lymph nodes, accompanied by enlargement of the liver and spleen, as well as systemic symptoms such as fever, night sweats, lethargy, and itchy skin, which may involve organs and tissues throughout the body. There are many classifications of lymphoma, which can be broadly divided into 2 groups: Hodgkin lymphoma (HL) and non-Hodgkin lymphoma (NHL). Of these, non-Hodgkin lymphoma has the highest incidence and is a highly aggressive, heterogeneous malignant tumor, whereas diffuse large B-cell lymphoma (DLBCL) is derived from mature B lymphocytes and is the most common type of non-Hodgkin lymphoma.^[1]^ Diffuse large B-cell lymphoma (DLBCL), derived from mature B lymphocytes, is the most common type of NHL,^[1]^ highly aggressive in clinical course and morphobiology, and is characterized by poor prognosis, recurrence and poor outcome.

The adrenal gland is one of the important endocrine organs of the body, consisting of the medulla and the cortex. Their structures and functions are distinct and interrelated. The adrenal glands maintain the development and growth of the body, the balance of water, electrolyte and energy metabolism, and the stability of blood pressure. Adrenal tumors usually result in a variety of physiological abnormalities. Most adrenal tumors are benign and primary adrenal malignancies (adrenocortical carcinoma, primary adrenal lymphoma) are relatively rare. Primary adrenal lymphoma (PAL) is a very rare and highly aggressive malignant tumor of unknown etiology, with atypical clinical symptoms and nonspecific imaging manifestations.^[2]^

## 2. Case report

**Figure 1.**
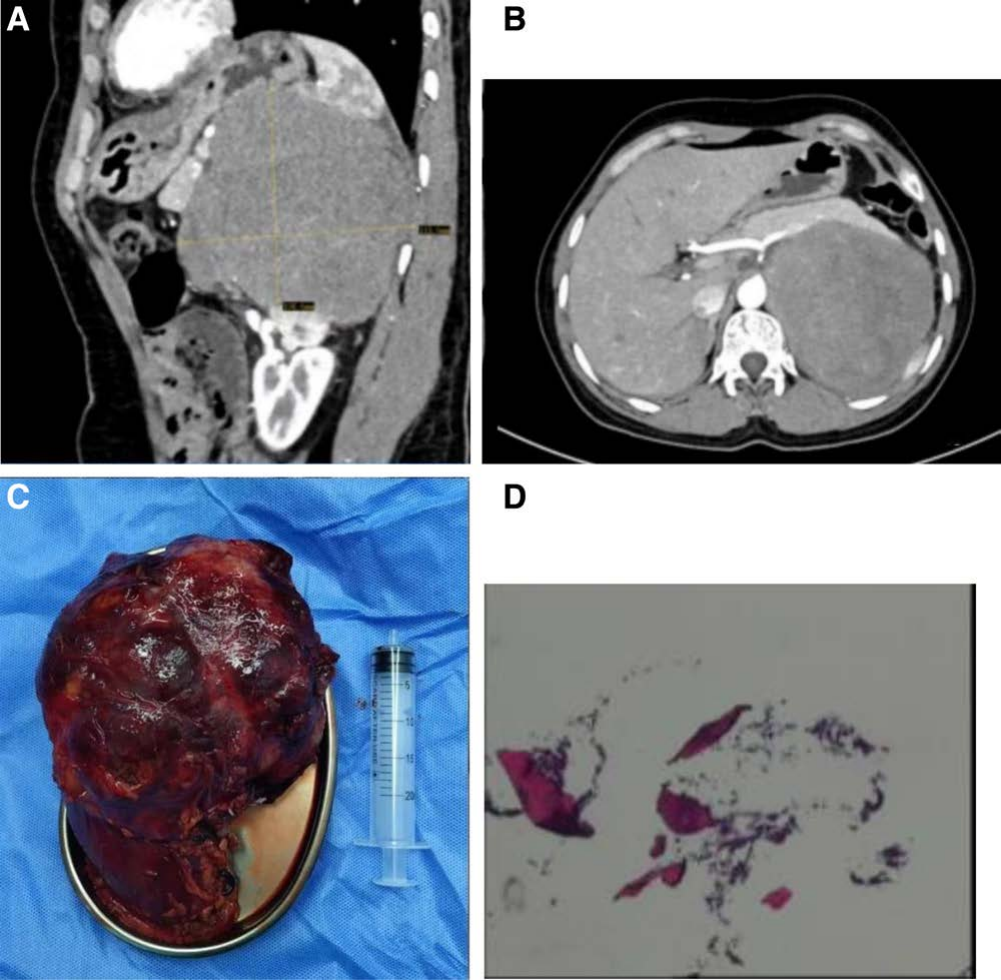
(A and B) Abdominal enhanced CT showed that the right adrenal nodule and the left adrenal region were huge space-occupying lesions, and pheochromocytoma was possible. (C) Postoperative adrenal tumor. (D) Pathological section after operation showed lymphoma.

A 42-year-old middle-aged woman was admitted to the hospital with a 1-year-old bilateral adrenal mass and 1-month-old left upper abdominal pain. The patient denied previous symptoms of hypertension and weakness of limbs. Enhanced CT of the abdomen showed a right adrenal nodule and a large space-occupying lesion in the left adrenal region, with a high likelihood of pheochromocytoma (Fig. 1A and B), and blood tests showed that aldosterone and cortisol were in the normal range in the standing and lying positions, and catecholamines were normal. Intraoperatively, a huge tumor was found in the left adrenal region, measuring about 12*12*10 cm (Fig. 1C), with infiltrative growth with the left kidney, spleen, and pancreatic tail, and the lymph nodes around the superior mesenteric artery and renal artery were obviously enlarged. Considering that the tumor had metastasized to the left kidney, spleen and pancreas, the left adrenal gland, spleen, left kidney and pancreatic tail were surgically removed. Postoperative pathology: lymphocytes were found in the kidney and lymphocytes in the pancreas; lymphocytes were found in the spleen. It is considered to be a lymphohematopoietic system tumor, most likely a lymphoma (Fig. 1D). Immunohistochemistry: tumor cells CD20(+), CD19(+), BCL-2 about 90%, C-MYC about 70%(+), CD10(−), MUM-1(+), BCL-6(+), CK(−), CD3(−), CyclinD1(−), CD30(−), CR(−), Syn(−), S-100(−), Syn(−), S-100(−) and Ki67 about 90% (+). Bone marrow smear showed markedly active proliferation of granulocytes. Bone marrow flow cytometry: no obvious abnormal B-lymphocyte-associated immunophenotypes were detected. Testing for MYC, BCL-2 and BCL-6 genes in the left nephrectomy sample was positive for BCL-6. Subsequently, the patient received R-CHOP chemotherapy with the following regimen: rituximab injection 560 mg + cyclophosphamide 1100 mg + epirubicin 100 mg + vincristine 2 mg + dexamethasone 15 mg. Three weeks later, chemotherapy with zebutinib + R-CHOP regimen was administered. The disease improved significantly.

## 3. Discussion

Primary diffuse large B-cell lymphoma of the adrenal gland (PADLBCL) is a rare malignant tumor with a poor prognosis, and its pathogenesis is unclear. One view is that it originates from the hematopoietic tissue of the adrenal gland,^[3]^ which requires further investigation. The clinical symptoms of primary diffuse large B-cell lymphoma (PADLB) are varied and not clearly specific.^[4]^ Symptoms such as fever, night sweats, and weight loss may be present, as well as localized symptoms such as abdominal and low back pain. Symptoms of adrenocortical insufficiency such as vomiting, malaise, skin pigmentation and hypotension may also be present. A minority of patients may be asymptomatic and be discovered accidentally during physical examination or screening for other diseases.^[5]^ DLBCL has no significant specificity in conventional imaging. Ultrasound, CT and MRI can show adrenal space-occupying lesions, but qualitative identification is difficult. Ultrasound is noninvasive, inexpensive and can show homogeneous hypoechoic masses. Currently, it has a limited diagnostic scope and can only be used for initial screening.^[6]^ The CT findings were basically similar to those previously reported in the literature, including tumor size, slightly lower density on CT plain scan, mild to moderate enhancement, uneven density, and adjacent infiltration. Among them, a few patients presented with necrosis or cysts, which were misdiagnosed as pheochromocytoma or ganglion cell tumor at the initial diagnosis, which is consistent with other reports.^[7]^ Therefore, it is difficult to diagnose PAL during the initial evaluation of conventional impact findings. Primary diffuse large B lymphoma of the adrenal gland is a rare adrenal malignancy. Currently, there are few domestic and international reports on it, and there is no consensus on its treatment. At present, the treatment options mainly refer to the treatment options for lymphoma. There are mainly the following treatment options: chemotherapy, surgery combined with chemotherapy, surgery alone, radiotherapy, autologous stem cell transplantation, and prophylactic intracavitary injection in the central nervous system.^[8]^ Currently, the first-line treatment option for PADLBCL is R-CHOP (rituximab + cyclophosphamide + cytarabine + vincristine + prednisone).^[9]^ Early studies showed that the 1-year survival rate of PADLBCL was close to 20%.^[10]^ With the use of new chemotherapeutic regimens, more patients have been treated with rituximab in a recent series of studies, with 2-year survival rates approaching 62%.^[11]^ Previous literature reported that surgery is one of the therapeutic options for adrenal lymphoma and one of the single factors affecting its prognosis.^[12]^ On the one hand, it can remove the tumor and reduce the tumor load, and on the other hand, it can obtain sufficient pathological information to provide direction and basis for the next step of treatment. For primary lymphoma, radical surgical treatment plus chemotherapy is preferred if there are no obvious contraindications to surgery.

In summary, PADLBCL has the characteristics of strong aggressiveness, poor prognosis, easy recurrence, poor efficacy and difficult diagnosis. Clinicians should consider the possibility of primary adrenal diffuse large B lymphoma when encountering huge adrenal space-occupying lesions, multidisciplinary consultation and diagnosis, and chemotherapy can be considered to reduce the tumor volume before surgery. Reduction of tumor size before surgery is a more favorable treatment. This article, as a case report, will provide data to support the diagnosis and treatment of PADLBCL in preparation for a large case study.

## Author contributions

**Conceptualization:** Yong Ou.

**Data curation:** Yong Ou.

**Investigation:** Kai Wang, Ting-Yu Jia, Hua-Kang Wang.

**Methodology:** Kai Wang, Ting-Yu Jia, Hua-Kang Wang, Hou-Chuan Chen.

**Project administration:** Zhi-Gang Chen.

**Resources:** Zhi-Gang Chen.

**Supervision:** Hua Yang.

**Validation:** Zhi Liao.

**Visualization:** Ming-Xian Chen.

**Writing – original draft:** Yong Ou.

**Writing – review & editing:** Zhong Zheng.
